# Rapid Eye Movement Sleep Behavior Disorder as a Prodromal Manifestation of Alpha-Synucleinopathies

**DOI:** 10.7759/cureus.117597

**Published:** 2026-10-07

**Authors:** Nimmi Ravindranath, Abid Bhat, Ashraf Gohar

**Affiliations:** 1 Sleep Medicine, University of Missouri-Kansas City, Kansas City, USA; 2 Pulmonary, Critical Care and Sleep Medicine, University of Missouri-Kansas City, Kansas City, USA

**Keywords:** alpha synucleinopathy, clonazepam, neuro-degeneration, neuro-immunology, parkinson' s disease, phenoconversion, prodromal neurodegeneration, rapid eye movement sleep behaviour disorder, rbd, rem sleep behavior disorder

## Abstract

Rapid eye movement (REM) sleep behavior disorder (RBD) is a parasomnia in which patients physically act out their dreams due to loss of the normal muscle paralysis that occurs during REM sleep. It is now recognized that isolated RBD (iRBD) is strongly associated with future development of α-synuclein-related neurodegenerative diseases, including Parkinson disease (PD), dementia with Lewy bodies (DLB), and multiple system atrophy (MSA). It is seen that most patients with polysomnography-confirmed iRBD eventually develop a clinically defined synucleinopathy, often after a long symptom-free interval. This long prodromal phase makes iRBD a unique clinical model for studying the earliest stages of neurodegeneration, before the onset of clear motor or cognitive disability. α-synuclein pathology frequently begins in the lower brainstem and autonomic nervous system, affecting REM sleep regulatory circuits early in the disease process. As pathology spreads, dopaminergic, cholinergic, and cortical systems become involved, leading to the gradual emergence of motor, autonomic, and cognitive symptoms. As a result, many individuals with iRBD exhibit subtle non-motor features such as hyposmia, constipation, orthostatic hypotension, anxiety, and mild cognitive changes years before a formal neurological diagnosis is made. Recent advances have improved the ability to identify patients at the highest risk of near-term phenoconversion. Quantitative measures of REM sleep without atonia, dopamine transporter imaging, autonomic testing, and emerging molecular biomarkers, including α-synuclein seed amplification assays, have shown promise in risk stratification. In addition, cognitive changes may occur earlier than previously recognized, particularly in patients with iRBD who also demonstrate hyposmia or dopaminergic dysfunction. Management of RBD remains largely symptomatic and focuses on injury prevention and sleep stabilization. While clonazepam and melatonin remain first-line therapies, alternative treatments including sodium oxybate, serotonergic agents, cannabinoids, and modulation of the gut-brain axis through probiotic supplementation have also been explored. This narrative review summarizes current knowledge regarding the clinical features, mechanisms, biomarkers, and management of RBD as a prodromal manifestation of α-synucleinopathies, with emphasis on its importance for early diagnosis and future disease-modifying research.

## Introduction and background

Rapid eye movement (REM) sleep behavior disorder (RBD) is a parasomnia in which patients physically act out their dreams due to loss of the normal muscle paralysis that occurs during REM sleep. RBD is broadly categorized into isolated (formerly idiopathic) RBD (iRBD) and secondary RBD. iRBD is defined by the absence of a clinically manifest neurological disorder, while secondary RBD occurs in association with underlying conditions, including neurodegenerative diseases, narcolepsy, focal brainstem lesions, antidepressant use, autoimmune or paraneoplastic disorders, substance withdrawal, and, less commonly, pediatric or developmental conditions [1]. RBD has also been reported in association with progressive supranuclear palsy, frontotemporal dementia, amyotrophic lateral sclerosis, spinocerebellar ataxia type 3, Huntington disease, and myotonic dystrophy type 2.

Although RBD was once considered a relatively benign sleep disorder, increasing evidence over the past two decades has shown that it is strongly associated with future development of alpha-synuclein-related neurodegenerative diseases, including Parkinson disease (PD), dementia with Lewy bodies (DLB), and multiple system atrophy (MSA) [1,2]. Large longitudinal studies have demonstrated that most patients with polysomnography-confirmed iRBD eventually develop a clinically defined synucleinopathy, often after a long symptom-free interval. Phenoconversion, referring to the transition from iRBD to a clinically manifest neurodegenerative disorder such as PD, occurs at an estimated annual rate of approximately 6-7%, with more than 70% of individuals converting within 12 years [2].

This long prodromal phase makes iRBD a unique clinical model for studying the earliest stages of neurodegeneration, before the actual onset of clear motor or cognitive disability. Neuropathological and imaging studies suggest that alpha-synuclein pathology frequently begins in the lower brainstem and autonomic nervous system, affecting REM sleep regulatory circuits early in the disease process [1,3]. As the pathology spreads, dopaminergic, cholinergic, and cortical systems become involved, leading to the gradual emergence of motor, autonomic, and cognitive symptoms [4,5]. As a result, many individuals with iRBD exhibit subtle non-motor features such as hyposmia, constipation, orthostatic hypotension, anxiety, and mild cognitive changes years before a formal neurological diagnosis is made [1,2].

Recent advances have improved the ability to identify patients at the highest risk of near-term phenoconversion. Quantitative measures of REM sleep without atonia, dopamine transporter imaging, autonomic testing, and emerging molecular biomarkers, including alpha-synuclein seed amplification assays, have shown promise in future risk stratification [6]. Additionally, growing evidence suggests that cognitive changes may occur earlier than previously recognized, particularly in patients with iRBD who also demonstrate hyposmia or dopaminergic dysfunction [5].

Management of RBD remains largely symptomatic and focuses on injury prevention and sleep stabilization. While clonazepam and melatonin remain first-line therapies [7], recent studies have explored alternative treatments such as sodium oxybate [8], serotonergic agents [9], cannabinoids [10], and modulation of the gut-brain axis through probiotic supplementation [11].

This narrative review summarizes current evidence regarding the clinical features, neurophysiological mechanisms, phenoconversion, biomarkers, and management of RBD as a prodromal manifestation of alpha-synucleinopathies, with particular emphasis on early identification, risk stratification, and future disease-modifying strategies. A literature search was conducted using PubMed and Google Scholar to identify relevant studies on iRBD, including its pathophysiology, biomarkers, phenoconversion, and management. Relevant original studies, reviews, and guidelines were included based on their relevance to the scope of this review. As a narrative review, no formal statistical synthesis or meta-analysis was performed.

## Review

Clinical presentation and natural history 

RBD typically presents with behaviors such as shouting, punching, kicking, or falling out of bed, most often occurring during the later part of the night when REM sleep predominates. Patients frequently recall vivid dreams that closely match their observed behaviors. Symptoms may fluctuate over time and often go unrecognized for years, particularly in individuals who sleep alone. Injuries to patients and bed partners are common. Differential diagnoses include non-REM parasomnias, sleep-related hypermotor epilepsy, periodic limb movement disorder, severe obstructive sleep apnea, sleep terrors arising from slow-wave sleep, benzodiazepine-induced automatism with amnesia, and nocturnal dissociative (psychogenic) behaviors [1].

Beyond nocturnal behaviors, individuals with iRBD often exhibit subtle daytime features that reflect early neurodegeneration. These may include reduced sense of smell, constipation, urinary dysfunction, orthostatic hypotension, anxiety, depression, and mild cognitive difficulties [1,2]. Subtle motor abnormalities may also be present years before a formal diagnosis of PD or DLB, supporting the view that iRBD represents an early multisystem disease state rather than an isolated sleep disorder. The temporal sequence of these prodromal manifestations is heterogeneous; olfactory and autonomic abnormalities may precede or accompany recognized dream-enactment behaviors by years, whereas cognitive and subtle motor abnormalities may become increasingly apparent closer to phenoconversion.

Neurophysiology: REM sleep without atonia and beyond 

REM sleep without atonia (RSWA), defined as the abnormal persistence of muscle activity during REM sleep when skeletal muscles are normally largely atonic, is the key neurophysiological feature of RBD, and it reflects dysfunction of brainstem circuits responsible for REM sleep-related muscle inhibition. These circuits include glutamatergic neurons in the sublaterodorsal nucleus and inhibitory GABAergic and glycinergic neurons in the medullary reticular formation [12]. Degeneration within these pathways leads to abnormal motor activity during REM sleep. A recently developed G51D alpha-synuclein fibril mouse model demonstrates that alpha-synuclein pathology within pontine REM-regulatory nuclei produces early REM sleep without atonia prior to motor decline, closely paralleling the temporal pattern seen in prodromal iRBD [13].

Recent studies have expanded understanding beyond standard polysomnography. Quantitative electromyography shows that the degree of RSWA may increase over time and correlate with disease progression. EEG studies suggest early cortical involvement, while abnormalities in transcranial magnetic stimulation and vestibular evoked myogenic potentials support widespread brainstem dysfunction even before clinical phenoconversion [12].

Epidemiology and diagnostic challenges 

Polysomnography-confirmed iRBD is relatively uncommon in the general population, whereas probable RBD identified through questionnaires is much more frequent [14]. This discrepancy reflects the limited accuracy of screening tools. Disorders that may mimic RBD include non-REM parasomnias, nocturnal seizures, obstructive sleep apnea with arousal-related movements, periodic limb movement disorder, medication-related behaviors, and nocturnal dissociative states [7]. Accurate diagnosis therefore requires overnight video-polysomnography.

RBD as a prodromal synucleinopathy 

*Pathophysiology: Where Synuclein Begins* 

Current evidence suggests that alpha-synuclein pathology often begins in the lower brainstem and autonomic nervous system, consistent with staging models of PD. Early involvement of REM sleep regulatory nuclei explains why RBD frequently precedes motor or cognitive symptoms by many years [1,3].

*Phenoconversion Rates* 

Large multicenter cohort studies demonstrate annual phenoconversion rates of approximately 6-7%, with more than 70% of patients converting within 12 years [2]. Lifetime conversion rates likely exceed 80%, highlighting the strong predictive value of iRBD.

*Predictors of Conversion* 

Factors associated with increased risk of phenoconversion include olfactory dysfunction, subtle parkinsonism, autonomic dysfunction, mild cognitive impairment, abnormal dopamine transporter imaging, and detection of alpha-synuclein in cerebrospinal fluid or peripheral tissue [5,6]. Cognitive impairment appears particularly relevant in predicting conversion to DLB rather than PD [5].

Hyposmia is one of the earliest and most prevalent prodromal markers of alpha-synucleinopathies, occurring in approximately two-thirds of individuals with isolated RBD and potentially preceding phenoconversion by more than 20 years. Standardized tests such as the Sniffin' Sticks [15] and University of Pennsylvania Smell Identification Test (UPSIT) [16] provide reliable assessment of olfactory dysfunction. Hyposmia demonstrates strong predictive value, with a hazard ratio exceeding that of other non-motor markers, and has been associated with cognitive decline, suggesting a role in predicting DLB. While it lacks specificity in distinguishing between different synucleinopathies and does not consistently worsen over time, its utility improves significantly when combined with other biomarkers, particularly dopamine transporter imaging, where it markedly enhances prediction of phenoconversion. Variability in olfactory dysfunction may be influenced by underlying neurogenesis and alpha-synuclein pathology [6].

Another potential early biomarker is impaired colour discrimination, typically developing after olfactory dysfunction but still preceding phenoconversion by over a decade. It is associated with an increased risk of progression to PD or DLB, with higher conversion rates observed in affected individuals. Although less extensively studied, retinal changes detected by optical coherence tomography, such as thinning of retinal layers, have also been linked to underlying neurodegeneration and may correlate with other prodromal markers. Together, these findings suggest that visual dysfunction may serve as a complementary diagnostic and prognostic indicator in iRBD, although further longitudinal validation is required [6]. The extent and consistency of follow-up may significantly impact phenoconversion detection, given that subtle parkinsonian and cognitive features often go unnoticed by patients and are revealed primarily on detailed clinical evaluation. Importantly, no marker reliably excludes phenoconversion, as a significant proportion of individuals with initially normal findings still develop disease over time [2].

Quantitative motor assessments show significant promise as early biomarkers in iRBD, with abnormalities detectable up to five to eight years before phenoconversion. In particular, upper extremity alternating-tap tests can distinguish affected individuals from healthy controls and serve as both prognostic and monitoring tools. A range of instrumental motor assessments, including gait analysis, speech evaluation, saccadic eye movements, rhythm testing, and finger tapping, demonstrate sensitivities and specificities of up to 80% in identifying iRBD. Gait studies reveal reduced speed, cadence, and decreased step variability, along with impaired postural control during dual-tasking, suggesting combined motor and cognitive dysfunction. Speech analysis shows features such as monopitch, prolonged pauses, and reduced fluency, reflecting early hypokinetic changes. Additionally, impairments in rhythm perception and finger tapping indicate prodromal bradykinesia, while increased antisaccadic errors suggest early executive dysfunction. Emerging approaches, including smartphone-based composite motor assessments, have demonstrated high diagnostic accuracy, with sensitivities up to 85% and specificities up to 92%, highlighting their potential for scalable, real-world monitoring [6].

Autonomic dysfunction is a common and early feature of iRBD, affecting cardiovascular, gastrointestinal, urinary, and sexual domains. Typically mild to moderate in severity, it lies between that seen in healthy individuals and patients with PD [6]. Certain symptoms, particularly constipation and erectile dysfunction, are associated with a higher risk of phenoconversion [2,6]. Objective measures, including heart rate variability, cardiac metaiodobenzylguanidine scintigraphy, and autonomic reflex testing, demonstrate early involvement of sympathetic and parasympathetic systems and support its role as a diagnostic biomarker [6].

Although phenoconversion rates are consistently high across cohorts, considerable heterogeneity exists in clinical outcomes. While some individuals develop PD with predominant motor manifestations, others progress to DLB or multiple system atrophy [2,6]. The biological factors determining these divergent trajectories remain incompletely understood and represent an important area of ongoing research [6].

Biomarkers 

Several biomarkers have shown promise in identifying individuals with iRBD who are at highest risk of phenoconversion. Emerging EEG-based markers (e.g., lower cyclic alternating pattern rates) and advanced analytical approaches, including machine learning models, show promise in improving diagnostic accuracy and predicting disease progression, although many remain investigational [6].

Biofluid biomarkers, particularly cerebrospinal fluid (CSF) markers, are promising for early detection of alpha-synucleinopathies. CSF-based real-time quaking-induced conversion (CSF RT-QuIC) detects pathological alpha-synuclein with 90-100% sensitivity and 90-98% specificity, with positivity associated with increased phenoconversion risk. Less invasive olfactory mucosa sampling shows lower sensitivity, while blood-based markers remain less reliable. Exosomal alpha-synuclein findings are inconsistent but may help differentiate disease subtypes. Serum neurofilament light chain and circulating microRNAs (e.g., miR-19b) may also indicate neuronal damage or phenoconversion. Overall, CSF RT-QuIC has the strongest evidence, while peripheral biomarkers require further validation [6,17].

Imaging biomarkers provide evidence of early neurodegeneration in iRBD, particularly nigrostriatal dopaminergic dysfunction. Dopamine transporter imaging with I-FP-CIT SPECT is the most established modality, with abnormalities predicting increased phenoconversion risk, especially when combined with cognitive and autonomic impairment. Fluorine-18 fluorodeoxyglucose (F-FDG) PET may identify metabolic patterns associated with disease progression and alpha-synucleinopathy subtypes. MRI demonstrates substantia nigra changes, cortical thinning, and altered connectivity associated with motor and cognitive deficits, although its prognostic value requires further validation. Overall, dopamine transporter imaging remains the strongest early imaging marker, while PET and MRI have promising but evolving roles [6].

Detection of phosphorylated alpha-synuclein in peripheral tissues represents a promising early biomarker of alpha-synucleinopathies. While earlier approaches using colonic and salivary gland biopsies showed limited sensitivity, skin biopsy has emerged as a less invasive and more reliable technique, demonstrating high specificity (approximately 100%) and moderate-to-high sensitivity (approximately 58-87%) in patients with iRBD [18-20]. Importantly, alpha-synuclein deposition can be detected even before dopaminergic imaging abnormalities, supporting its role as an early indicator of neurodegeneration. Although primarily a diagnostic biomarker, its prognostic value and ability to predict phenoconversion require further longitudinal validation [6].

Genetic factors represent a potential early indicator of phenoconversion risk in iRBD, although its genetic architecture differs from that of PD and other alpha-synucleinopathies. Most established risk variants for PD and DLB, such as *LRRK2* and *APOE* epsilon4, are not associated with iRBD, highlighting a distinct genetic background. In contrast, *GBA* variants are present in approximately 10% of patients with iRBD and have been associated with increased phenoconversion risk, with severe variants potentially associated with more rapid phenoconversion [21]. Additionally, variants in the SNCA locus may influence both susceptibility and rate of progression, with evidence suggesting that different regions of the gene are implicated in iRBD compared with PD. Although these findings support the role of genetic markers as prognostic indicators, their clinical utility remains limited, and further large-scale and longitudinal studies are required to validate their predictive value [6].

Wearable technologies are emerging as a promising adjunctive tool for RBD detection. Recent studies using wrist-worn actigraphy combined with machine learning have demonstrated good accuracy in identifying characteristic nocturnal movement patterns associated with RBD. Although video-polysomnography remains the diagnostic gold standard, these approaches may facilitate large-scale screening and longitudinal monitoring of at-risk individuals [22].

Management strategies 

Management of RBD focuses on reducing injury risk and improving sleep quality. Environmental safety modifications remain a cornerstone of RBD management. Pharmacologic treatment commonly includes immediate-release melatonin or clonazepam, consistent with recommendations from the American Academy of Sleep Medicine. Melatonin is often preferred due to its favorable side-effect profile. Immediate-release melatonin may be initiated at 3 mg at bedtime and titrated in 3-mg increments up to 15 mg based on clinical response. If symptoms are not adequately controlled, clonazepam may be considered; most patients initially respond to doses of 0.25-1.0 mg at bedtime [7].

Furthermore, there are a few emerging therapies under investigation. Sodium oxybate has shown potential benefit in treatment-resistant RBD, although placebo effects are substantial [8]. Transdermal rivastigmine may reduce dream enactment behaviors in patients with RBD and mild cognitive impairment and in patients with RBD secondary to Parkinson disease. Rivastigmine is typically administered as a transdermal patch, starting at 4.6 mg/24 hours and titrated up to 13.3 mg daily as tolerated. Pramipexole may also improve RBD symptoms, particularly in patients with increased periodic limb movements on polysomnography, possibly through reduction of ancillary motor activity or an underlying sleep-fragmenting periodic limb movement disorder. Evidence for other agents, including donepezil and levodopa, remains limited or inconclusive. Deep brain stimulation has not demonstrated clinically significant improvement in RBD symptoms, and current AASM guidelines suggest against its use specifically for treatment of secondary RBD [7].

Cannabidiol has not consistently demonstrated efficacy so far [10]. Serotonergic agents such as 5-hydroxytryptophan may improve sleep stability rather than core RBD behaviors [9]. A small pilot study has explored probiotic supplementation in patients with PD and RBD; however, the evidence remains preliminary and is insufficient to support probiotic supplementation as an established treatment for RBD [11].

Exercise has demonstrated benefits for motor function, sleep quality, cognition, and quality of life in PD and related neurodegenerative disorders. However, direct evidence supporting exercise as a treatment for iRBD remains limited. Although experimental evidence suggests potential effects on neuroinflammation, mitochondrial function, and alpha-synuclein-related pathways, whether exercise modifies the risk of phenoconversion in iRBD remains unknown [23].

At present, treatment strategies remain primarily symptomatic and do not alter the underlying neurodegenerative process. However, recognition of iRBD as a prodromal stage of synucleinopathy has generated considerable interest in disease-modifying therapies. Future clinical trials may enrol patients with iRBD as an ideal population for testing interventions aimed at delaying or preventing phenoconversion. The ability to identify individuals at high risk of future neurodegenerative disease also raises important ethical considerations. Clinicians must balance the potential benefits of early diagnosis with the psychological impact of informing patients about their increased risk of PD or dementia. As predictive biomarkers become increasingly accurate, the development of standardized counseling approaches may become an important component of clinical practice.

## Conclusions

Isolated RBD represents one of the most robust prodromal markers of alpha-synuclein-related neurodegenerative disorders, including PD, DLB, and multiple system atrophy. Its prolonged preclinical phase and high likelihood of phenoconversion provide a unique window to study early disease mechanisms before the onset of irreversible neurodegeneration. A growing body of evidence supports the role of multimodal biomarkers, including neurophysiological, motor, olfactory, autonomic, imaging, biofluid, and genetic markers, in improving early detection, risk stratification, and prediction of disease trajectory. Among these, dopamine transporter imaging and CSF-based assays demonstrate particularly strong diagnostic and prognostic potential, while other markers continue to evolve. However, no single biomarker is sufficient to predict phenoconversion with certainty, highlighting the need for integrated, multimodal approaches.

Advances in the understanding of underlying pathophysiology and biomarker development may enable earlier identification of high-risk individuals and facilitate the implementation of disease-modifying therapies. Ultimately, iRBD offers a critical opportunity for early intervention and the design of clinical trials aimed at delaying or preventing the progression to overt alpha-synucleinopathies. Emerging developments in genetics, wearable technologies, and lifestyle-based interventions continue to expand the conceptual framework of iRBD. Future approaches will likely combine molecular biomarkers, neuroimaging, wearable technologies, and individualized risk profiling to identify patients most likely to benefit from disease-modifying therapies. Such multimodal strategies may ultimately transform iRBD from a predictive marker of neurodegeneration into a practical target for early intervention.
